# Clinical Features and Plasma Metabolites Analysis in Obese Chinese Children With Nonalcoholic Fatty Liver Disease

**DOI:** 10.1210/jendso/bvaf032

**Published:** 2025-02-24

**Authors:** Xiaoxiao Liu, Shifeng Ma, Jing Li, Mingkun Song, Yun Li, Yingyi Qi, Fei Liu, Zhongze Fang, Rongxiu Zheng

**Affiliations:** Department of Pediatrics, Tianjin Medical University General Hospital, Tianjin 300000, China; Department of Pediatrics, Tianjin Medical University General Hospital, Tianjin 300000, China; Department of Epidemiology and Biostatistics, School of Public Health, Tianjin Medical University, Tianjin 300000, China; Tianjin Key Laboratory of Environment, Nutrition and Public Health, Tianjin 300000, China; Tianjin Center for International Collaborative Research on Environment, Nutrition and Public Health, Tianjin 300000, China; Department of Pediatrics, Tianjin Medical University General Hospital, Tianjin 300000, China; Department of Pediatrics, Tianjin Medical University General Hospital, Tianjin 300000, China; Department of Pediatrics, Tianjin Medical University General Hospital, Tianjin 300000, China; Department of Pediatrics, Tianjin Medical University General Hospital, Tianjin 300000, China; Tianjin Key Laboratory of Environment, Nutrition and Public Health, Tianjin 300000, China; Tianjin Center for International Collaborative Research on Environment, Nutrition and Public Health, Tianjin 300000, China; Department of Toxicology and Sanitary Chemistry, School of Public Health, Tianjin Medical University, Tianjin 300000, China; Department of Pediatrics, Tianjin Medical University General Hospital, Tianjin 300000, China

**Keywords:** nonalcoholic fatty liver disease, plasma metabolomics, machine learning, pediatric obesity

## Abstract

**Objective:**

This study aimed to investigate the clinical characteristics and plasma metabolites of nonalcoholic fatty liver disease (NAFLD) in obese Chinese children and to develop machine learning-based NAFLD diagnostic models.

**Methods:**

We recruited 222 obese children aged 4 to 17 years and divided them into an obese control group and an obese NAFLD group based on liver ultrasonography. Mass spectrometry metabolomic analysis was used to measure 106 metabolites in plasma. Binary logistic regression was used to identify NAFLD-related clinical variables. NAFLD-specific metabolites were illustrated via volcano plots, cluster heatmaps, and metabolic network diagrams. Additionally, we applied 8 machine learning methods to construct 3 diagnostic models based on clinical variables, metabolites, and clinical variables combined with metabolites.

**Results:**

By evaluating clinical variables and plasma metabolites, we identified 16 clinical variables and 14 plasma metabolites closely associated with NAFLD. We discovered that the level of 18:0 to 22:6 phosphatidylethanolamines was positively correlated with the levels of total cholesterol, triglyceride-glucose index, and triglyceride to high-density lipoprotein cholesterol ratio, whereas the level of glycocholic acid was positively correlated with the levels of alanine aminotransferase, gamma-glutamyl transferase, insulin, and the homeostasis model assessment of insulin resistance. Additionally, we successfully developed 3 NAFLD diagnostic models that showed excellent diagnostic performance (areas under the receiver operating characteristic curves of 0.917, 0.954, and 0.957, respectively).

**Conclusions:**

We identified 16 clinical variables and 14 plasma metabolites associated with NAFLD in obese Chinese children. Diagnostic models using these features showed excellent performance, indicating their potential for diagnosis.

Nonalcoholic fatty liver disease (NAFLD) is characterized by significant lipid deposits in the liver parenchyma without a history of excessive alcohol consumption. Obese children are more likely to have NAFLD, with 27.8% to 41.2% morbidity [1-3]. With an increasing prevalence of obesity, NAFLD has become the most common cause of chronic liver disease in children and adolescents [4, 5]. It is a continuous liver condition ranging from simple fatty liver to nonalcoholic steatohepatitis and cirrhosis. Abnormal lipid metabolism contributes to this progression [6]. Approximately 10% patients with simple fatty liver may develop into steatohepatitis and cirrhosis [7].

Plasma lipidomic analysis has revealed that fatty acids, phosphatidylethanolamines (PE), phosphatidylcholines (PC), diacylglycerols, and triacylglycerols are key factors in the development of NAFLD [8]. Bile acids (BAs) also act as specific signaling molecules to promote the absorption of dietary lipids and fat-soluble vitamins, potentially playing a role in the pathogenesis of NAFLD [9].

Diagnosing NAFLD in children mostly relies on imaging tests and serum biomarkers. But magnetic resonance spectroscopy is costly and computed tomography is radiative. Ultrasound is often used but varies in sensitivity [10]. Biomarkers, such as alanine aminotransferase (ALT) and aspartate aminotransferase (AST), are not sensitive or specific for NAFLD [11]. Metabolome biomarkers may improve diagnostic accuracy by revealing metabolic pathways related to NAFLD [12]. Machine learning can extract important relationships related to target categories by analyzing patterns in data sets [13]. In combination with clinical variables and metabolomics, machine learning offers a possible pathway for identifying unique combinations of metabolites that can identify NAFLD in obese children. The objective of this paper is to develop a new model for diagnosing NAFLD in obese children using machine learning by analyzing clinical variables and metabolite characteristics.

## Methods

### Participants

Participants were recruited from Tianjin Medical University General Hospital from October 2020 to June 2024. The inclusion criteria for the obese NAFLD (ON) group were (1) aged 4 to 17 years; (2) body mass index (BMI) > 95th percentile for age and gender [14]; (3) NAFLD diagnosed by liver ultrasound [15]; (4) no history of alcohol consumption; and (5) exclusion of other diseases that can cause obesity and fatty liver. The inclusion criteria for the obese control (OC) group were (1) aged 4 to 17 years; (2) BMI >95th percentile for age and gender [14]; (3) NAFLD excluded by liver ultrasound; (4) no history of alcohol consumption; and (5) exclusion of other diseases that can cause obesity and fatty liver. Every participant underwent abdominal liver ultrasound test using the same ultrasound scanner (Philips HD11). The diagnosis of NAFLD was based on the NAFLD diagnosis and management guidelines proposed by Chinese National NAFLD Consensus Workshop [15].

The study protocol was approved by the Ethics Committee of Tianjin Medical University General Hospital.

### Anthropometric and Biochemical Data

Clinical assessments and measurements were performed by experienced pediatricians. Body weight, height, systolic blood pressure (SBP), diastolic blood pressure, and acanthosis nigricans were recorded. BMI was calculated for every participant. BMI *Z*-score was recorded according to Chinese criteria [16]. Waist circumference (WC) was measured at the midpoint between the lower edge of the last palpable rib and the top of the iliac crest via nonstretchy tape. The waist-to-height ratio was calculated by WC (cm) to height (cm).

All blood sample parameters were drawn after an overnight fast. Fasting blood serum was used to measure total cholesterol (TC), fasting plasma glucose (FPG), insulin (INS), high-density lipoprotein cholesterol (HDL-C), triglyceride (TG), ALT, alkaline phosphatase, glycated hemoglobin A1c (HbA1c), low-density lipoprotein cholesterol (LDL-C), albumin, uric acid, AST, γ-glutamyl transferase (GGT), total bilirubin, and direct bilirubin. All participants underwent standard oral glucose tolerance test, as previously described [17]. In summary, every participant took an oral glucose solution containing 1.75 g/kg of body weight (up to 75 g of glucose), and blood samples were taken at 30, 60, 120, and 180 minutes to detect changes in blood glucose and insulin levels.

Homeostasis Model Assessment of Insulin Resistance (HOMA-IR) = FPG × INS/22.5, and whole-body insulin sensitivity index (WBISI) = 10 000/√FPG × INS × mean glucose × mean insulin [18]. Lipid-related indices, including the triglyceride-glucose index (TyG) and TG/HDL-C, were calculated via as follows: TyG = ln (TG (mg/dL) × FPG (mg/dL)/2); TG/HDL-C = TG (mg/dL)/HDL-C (mg/dL) [19].

### Serum Metabolites Measurement

A 3-mL fasting blood sample was centrifuged at 4 °C, plasma was separated from blood cells, and plasma was frozen at −80 °C. A total of 75 lipid metabolites, 11 fatty acids, and 20 BAs in plasma were analyzed. All samples and reagents were prepared according to the manufacturers’ instructions. A total of 20 μL plasma, 20 μL mixed internal standard solution, and 20 μL water were added into an Eppendorf tube in sequence. The mixture was vortexed for 30 seconds, followed by addition of 200 μL chloroform-methanol extraction solution (2:1). After vortexing for an additional 20 seconds, the mixture was allowed to stand at 4 °C for 30 minutes. Then this was centrifuged at 12 500 rpm for 10 minutes and a 1-mL syringe was used to draw the lower liquid into a 0.5-mL EP tube. Next, 60 μL of the liquid was transferred into another EP tube and dried under nitrogen. After drying, the residue was reconstituted with 50 μL of acetonitrile-isopropyl alcohol and vortexed for 60 seconds before liquid chromatography-mass spectrometry analysis. Eksigent Ultra LC 100 was used in conjunction with the AB sciex Triple TOF 5600 MS detecting system to identify and quantify metabolites. Under the negative ion model, the separation was performed using a 2.1 × 100 mm XBridge polypeptide BEH C18 column (Waters) and a 4 × 2.0-mm protective column (Phenomenex). The raw data were acquired in negative ion mode and all results were expressed in nmol/L. The lipids in the samples were qualitatively analyzed via Peak View 1.2 software, MultiQuant 2.1 software, and calibrated according to the standard data.

### Statistical Analysis and Development of Interpretable Machine Learning Models for NAFLD

Categorical variables were presented as percentages, whereas continuous variables were presented as medians (P25, P75). The chi-squared test was used for categorical data. The Kruskal-Wallis test was used to analyze continuous clinical and metabolite variables.

Binary logistic regression was performed using 3 multivariate models, and odds ratios and 95% CIs were assessed for clinical variables (multivariable model 1 was adjusted for age, gender; multivariable model 2 was adjusted for age, gender, and BMI; multivariable model 3 was adjusted for age, gender, BMI and WBISI). Gender, age, and BMI were adjusted as confounding factors; restricted cubic spline models were used to elucidate the dose-response relationship between clinical variables (insulin sensitivity and lipid-related indicators) and NAFLD risk. Four percentiles of clinical variables (P25, P50, P75, and P95) were chosen as nodes, and restricted cubic spline curves for NAFLD occurrence and clinical variables were generated via R language 4.4.0.

NAFLD-specific metabolites, showing significant differences between OC and ON, were elucidated through volcano plots and cluster heatmaps, generated using GraphPad Prism 10.2.0 and MetaboAnalyst 5.0, respectively [20]. Significance was defined as a false discovery rate (FDR)-corrected *P* < .05. Chemical and biochemical relationships of NAFLD-specific metabolites were created by mapping onto MetaMapp and by visualization with Cytoscape 3.10.2 [21, 22]. Spearman correlation analysis was employed to determine the correlation coefficients and 2-tailed *P* between NAFLD-specific metabolites and clinical variables; Python 3.9 was used to generate correlation heatmaps.

Different machine learning methods were used to build and optimize the NAFLD machine learning models comprehensively. Logistic regression is used for baseline and interpretive binary classification; random forest and extreme gradient boosting handle complex data relationships through integrated learning; support vector machines and categorical boosting are good at processing high-dimensional data and classification features to provide high-precision prediction. Gaussian-naive Bayes and adaptive boosting provide efficient and robust classification to improve model accuracy [23]. We developed 3 diagnostic models for obese children with NAFLD based on clinical variables, metabolites, and clinical variables combined metabolites, respectively. The performance of each model was evaluated via metrics such as accuracy, sensitivity, specificity, precision, F1 score, and area under curve (AUC). To gain a deeper understanding of the inference mechanism of the random forest model, the Shapley additive explanation (SHAP) method from the SHAP package (https://github.com/slundberg/shap) was used for further analysis. These models were implemented in Python 3.9 via the scikit-learn package (version 1.5.1).

## Results

### Clinical Characteristics of Participants

A total of 222 obese Chinese children aged 4 to 17 years were included. A total of 118 in the OC group (53 boys and 65 girls), whose median age was 10.8 years, and 104 in the ON group (64 boys and 40 girls), whose median age was 11.7 years. The demographic characteristics, physical examination results, and biochemical parameters are summarized in Table 1. Compared to the OC group, there were more boys in the ON group (*P* < .05), and participants in the ON group were older (*P* < .05). BMI, BMI *Z* score, blood pressure, WC, waist-to-height ratio, and hip circumference of participants in the ON group were bigger than those in the OC group (*P* < .05). More participants had acanthosis nigricans in the ON group (*P* < .05). Additionally, many biochemical indicators in the 2 groups were different. Patients in the ON group had higher ALT, AST, and GGT, whereas ALP and LDH were similar in the 2 groups. Glucose metabolism indicators, HbA1c, INS, HOMA-IR, and WBISI were higher in the ON group, whereas fasting blood glucose was still similar in 2 groups. Lipid-related indicators tested in this study, TC, TG, HDL-C, LDL-C, TyG, and TG/HDL-C were all higher in the ON group. Uric acid was higher in the ON group. There was no difference between bilirubin levels in the 2 groups.

**Table 1.** Clinical characteristics of the study participants

Variables	OC group	ON group	*P*
Gender (M/F)	53/65	64/40	.013
Age (y)	10.8 (9.8, 12.7)	11.7 (10.5, 13.4)	.012
Father's BMI (kg/m^2^)	26.12 (24.65, 28.52)	26.08 (24.21, 28.64)	.678
Mather's BMI (kg/m^2^)	22.89 (21.22, 24.97)	24.13 (21.13, 27.34)	.014
BMI (kg/m^2^)	24.11 (21.39, 27.14)	27.41 (24.76, 29.74)	<.001
BMI *Z* score (SD)	2.83 (2.57, 3.20)	3.55 (2.73, 4.80)	<.001
SBP (mm Hg)	112 (109, 123)	118 (118, 126)	<.001
DBP (mm Hg)	71 (67, 77)	77 (70, 80)	<.001
WC (cm)	78.5 (70.0, 88.0)	86 (75.2, 98.0)	.002
WHtR (%)	0.51 (0.46, 0.57)	0.55 (0.49, 0.61)	.001
Hip (cm)	92.0 (85.0, 100.0)	100.5 (88.0, 109.7)	.004
Acanthosis nigricans	35 (29.7%)	58 (55.7%)	<.001
Biochemical indicators			
ALT (IU/L)	17 (13, 22)	28 (19, 51)	<.001
AST (IU/L)	21 (18, 25)	24 (20, 32)	.004
GGT (IU/L)	14 (12, 18)	21 (17, 30)	<.001
ALP (IU/L)	269 (206, 343)	262 (200, 327)	.318
LDH (U/L)	217 (197, 252)	226 (197, 258)	.862
FPG (mmol/L)	4.7 (4.5, 5.0)	4.8 (4.5, 5.2)	.200
INS (mU/L)	14.7 (10.3, 22.5)	23.5 (16.1, 33.9)	<.001
HbA1c (%)	5.4 (5.2, 5.5)	5.6 (5.4, 5.8)	<.001
HOMA-IR	3.01 (2.22, 4.57)	5.11 (3.31, 7.73)	<.001
WBISI	4.75 (2.42, 6.25)	2.76 (0.96, 4.85)	<.001
TC (mmol/L)	4.00 (3.60, 4.60)	4.20 (3.80, 4.80)	.008
TG (mmol/L)	1.02 (0.76, 1.36)	1.29 (0.96, 1.79)	<.001
HDL-C (mmol/L)	1.23 (1.06, 1.46)	1.11 (1.01, 1.23)	<.001
LDL-C (mmol/L)	2.30 (1.99, 2.73)	2.56 (2.18, 3.06)	.001
TyG	8.25 (7.95, 8.54)	8.55 (8.22, 8.88)	<.001
TG/HDL-C	1.31 (0.86, 1.81)	1.85 (1.23, 2.72)	<.001
URIC (umol/L)	349.5 (304.2, 445.5)	420 (351.0, 491.0)	<.001
TBIL (umol/L)	8.7 (6.9, 11.3)	9.0 (7.1, 11.6)	.725

Note: *P* was assessed via Mann-Whitney *U* test. Continuous variables are presented as the median (25th, 75th percentile).

Abbreviations: ALP, alkaline phosphatase; ALT, alanine aminotransferase; AST, aspartate aminotransferase; BMI, body mass index; DBP, diastolic blood pressure; FPG, fasting plasma glucose; GGT, γ-glutamyl transferase; HbA1c, glycosylated hemoglobin; HDL-C, high density lipoprotein cholesterol; HOMA-IR, homeostasis model assessment of insulin resistance; INS, insulin; LDH, lactate dehydrogenase; LDL-C, low-density lipoprotein cholesterol; OC, obese control group; ON, obese nonalcoholic fatty liver disease group; SBP, systolic pressure; TBIL, total bilirubin; TC, total cholesterol; TG, triglyceride; TG/HDL-C, ratio of triglycerides to high-density lipoprotein; TyG, triglyceride-glucose index; URIC, uric acid; WBISI, whole-body insulin sensitivity index; WC, waist circumference; WHtR, waist-to-height ratio.

**Figure 1.**
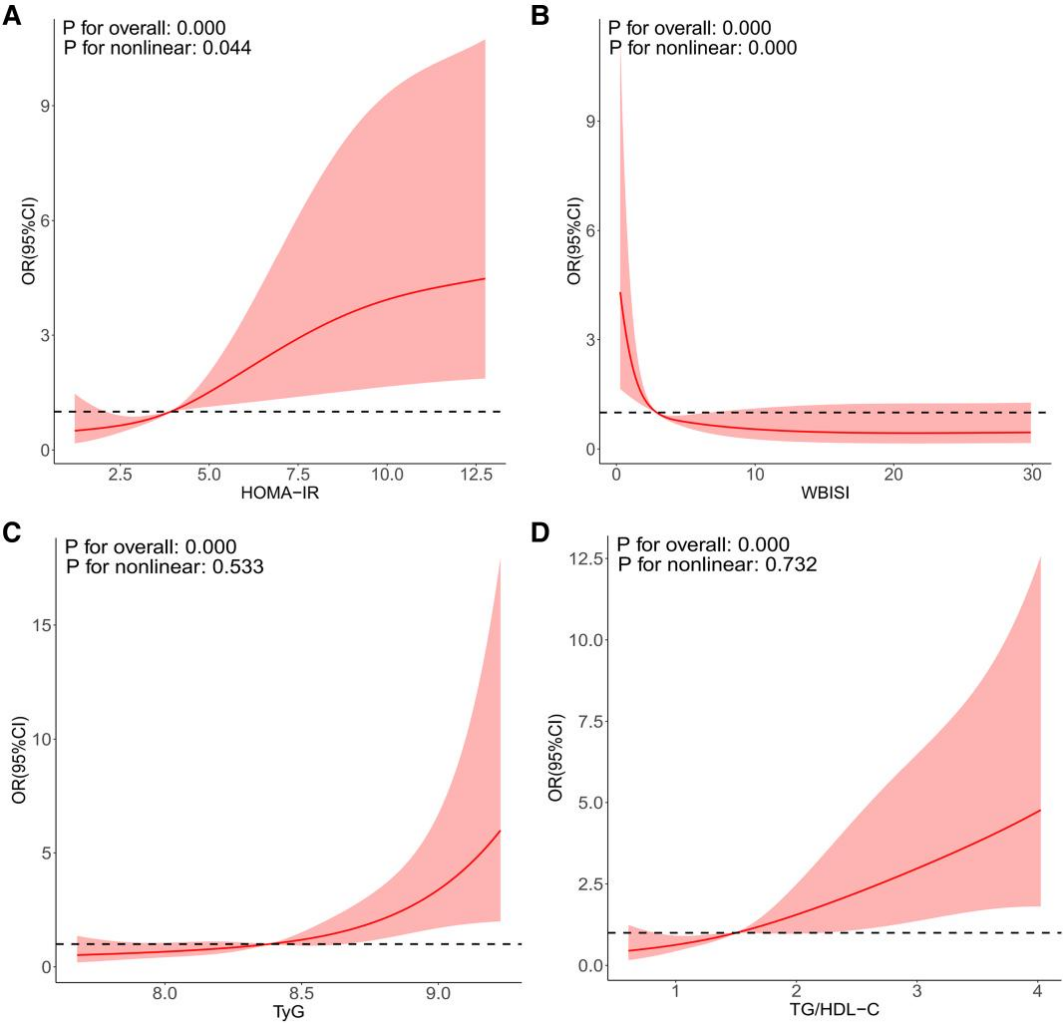
Dose-response relationship analysis of clinical variables with NAFLD risk in restrictive cubic spline models, adjusting for sex, age, and BMI as confounding factors. (A) Relationship between HOMA-IR and NAFLD. NAFLD incidence increases when the HOMA-IR grade is greater than 3.945. (B) Relationship between the WBISI and NAFLD. NAFLD incidence decreases when the WBISI is greater than 2.804. (C) Relationship between the TyG index and NAFLD. NAFLD incidence increases when TyG is greater than 8.386. (D) Relationship between TG/HDL-C and NAFLD risk. NAFLD risk increases when the TG/HDL-C is greater than 1.501.

Three kinds of multivariable binary logistic regression models revealed a correlation between 16 clinical variables and NAFLD (Table 2). After adjusting for age, gender, and BMI as confounding factors, we investigated the dose-response relationships between HOMA-IR, WBISI, TyG, TG/HDL-C, and the incidence of NAFLD. In the restricted cubic spline model, there was a nonlinear dose-response relationship between HOMA-IR and the incidence of NAFLD. When the HOMA-IR score was greater than 3.945, the risk of NAFLD increased (Fig. 1A). A nonlinear dose-response relationship was observed between WBISI and the incidence of NAFLD. When the WBISI exceeded 2.804, the risk of developing NAFLD decreased (Fig. 1B). In the restricted cubic spline model, there was a linear dose-response relationship between TyG, TG/HDL-C, and the incidence of NAFLD. The risk of NAFLD gradually increased as TyG and TG/HDL-C levels rose, with a particularly pronounced increase observed when TyG exceeded 8.386 (Fig. 1C) or TG/HDL-C surpassed 1.501 (Fig. 1D).

**Table 2.** Logistic regression analysis based on clinical variables

	Multivariable model 1		Multivariable model 2		Multivariable model 3	
	*P*	OR (95% CI)	*P*	OR (95% CI)	*P*	OR (95% CI)
SBP (mm Hg)	.002	1.041 (1.015, 1.067)	.010	1.035 (1.008, 1.063)	.013	1.034 (1.007, 1.062)
DBP (mm Hg)	.006	1.044 (1.012, 1.077)	.020	1.039 (1.006, 1.073)	.025	1.038 (1.005, 1.072)
Acanthosis nigricans	.001	0.372 (0.211, 0.654)	.010	0.453 (0.248, 0.827)	.050	0.964 (0.930, 1.000)
WC (mm)	.091	1.017 (0.997, 1.038)	.640	1.006 (0.982, 1.029)	.776	1.003 (0.98, 1.027)
Hip (mm)	.235	1.012 (0.993, 1.031)	.753	1.003 (0.983, 1.024)	.957	1.001 (0.980, 1.021)
WHtR (%)	.005	40.781 (3.029, 549.057)	.062	13.462 (0.873, 207.503)	.077	12.660 (0.757, 211.642)
BMI *Z* score (SD)	<.001	2.111 (1.510, 2.951)	<.001	2.352 (1.501, 3.688)	<.001	2.278 (1.454, 3.568)
ALT (IU/L)	<.001	1.024 (1.011, 1.037)	.002	1.021 (1.008,1.035)	.003	1.020 (1.006, 1.033)
AST (IU/L)	.010	1.029 (1.007, 1.051)	.030	1.025 (1.002, 1.048)	.037	1.024 (1.001, 1.047)
GGT (IU/L)	<.001	1.100 (1.057, 1.144)	<.001	1.093 (1.050, 1.138)	<.001	1.087 (1.044, 1.131)
URIC (µmol/L)	.006	1.004 (1.001, 1.007)	.050	1.003 (1.000, 1.006)	.114	1.002 (0.999, 1.005)
TC (mmol/L)	.001	2.125 (1.388, 3.254)	.001	2.046 (1.327, 3.155)	.001	2.107 (1.354, 3.279)
TG (mmol/L)	<.001	3.679 (2.049, 6.607)	<.001	3.357 (1.890, 5.962)	<.001	3.144 (1.735, 5.699)
HDL-C (mmol/L)	.001	0.144 (0.046, 0.453)	.003	0.173 (0.054, 0.555)	.011	0.215 (0.066, 0.701)
LDL-C (mmol/L)	<.001	2.527 (1.566, 4.076)	.001	2.272 (1.394, 3.703)	.002	2.207 (1.352, 3.603)
HbA1c (%)	<.001	22.869 (6.142, 85.151)	<.001	23.767 (6.232, 90.637)	<.001	21.940 (5.712, 84.279)
INS (mU/L)	<.001	1.040 (1.019, 1.062)	.002	1.034 (1.012, 1.056)	.010	1.030 (1.007, 1.053)
HOMA-IR	<.001	1.206 (1.094, 1.331)	.001	1.173 (1.064, 1.294)	.008	1.151 (1.037, 1.278)
TyG	<.001	5.186 (2.560, 10.505)	<.001	4.685 (2.312, 9.494)	<.001	4.480 (2.106, 9.530)
TG/HDL	<.001	2.122 (1.528, 2.946)	<.001	2.013 (1.460, 2.774)	<.001	1.930 (1.388, 2.685)
WBISI	.013	0.954 (0.919, 0.990)	.038	0.962 (0.928, 0.998)	—	—

Note: Multivariable model 1 was adjusted for age and gender. Multivariable model 2 was adjusted for age, gender, and BMI. Multivariable model 3 was adjusted for age, gender, BMI, and WBISI.

Abbreviations: ALT, alanine aminotransferase; AST, aspartate aminotransferase; BMI, body mass index; DBP, diastolic blood pressure; GGT, γ-glutamyl transferase; HbA1c, glycosylated hemoglobin; HDL-C, high density lipoprotein cholesterol; HOMA-IR, homeostasis model assessment of insulin resistance; INS, insulin; LDL-C, low-density lipoprotein cholesterol; OR, odds ratio; SBP, systolic pressure; TC, total cholesterol; TG, triglyceride; TyG, triglyceride-glucose index; URIC, uric acid; WBISI, whole-body insulin sensitivity index; WC, waist circumference; WHtR, waist-to-height ratio.

### Plasma Metabolite Characteristics

**Figure 2.**
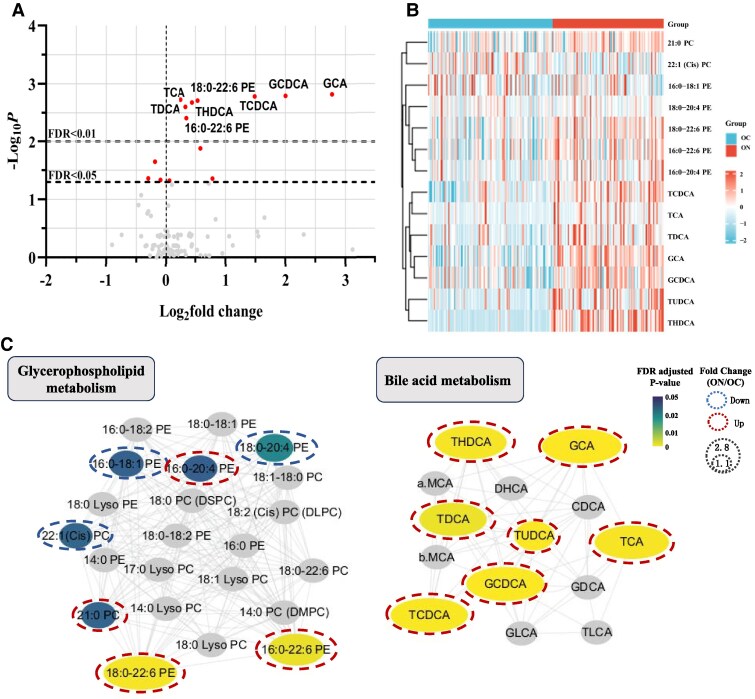
Specific metabolites in the ON group compared with the OC group. (A) Volcano plot and (B) heatmap of specific metabolites (NAFLD-specific metabolites, FDR-adjusted *P* < .05) between the ON and OC groups. In the volcano plot, significant metabolites are labeled. In the heatmap, the concentrations of each metabolite was standardized by the autoscaling of features, followed by metabolite clustering via the Ward method with Euclidean distance. (C) Relevance of NAFLD-specific metabolites in metabolic pathways. Biochemical and chemical networks of the selected significant metabolites indicated the pertinent metabolic pathways in NAFLD status. In the network, nodes representing NAFLD-specific metabolites are determined by FDR-adjusted *P*. Nodes of other selected metabolites with FDR-adjusted *P* > 0.05 are also included.

A total of 106 metabolites were detected in plasma, among which 14 were different between the OC group and the ON group (Fig. 2A and B, Supplementary Table S1) [24]. These included 5 PE metabolites (16:0-18:1 PE, 16:0-20:4 PE, 16:0-22:6 PE, 18:0-20:4 PE, and 18:0-22:6 PE), 2 PC metabolites (21:0 PC and 22:1(Cis)PC), and 7 BA metabolites (taurine conjugated cholic acid, tauroursodeoxycholic acid, taurohyocholic acid [THDCA], taurocholic acid, tauroursodeoxycholic acid [TDCA], glycocholic acid [GCA], and glycochenodeoxycholic acid).

We mapped NAFLD-specific metabolites and other selected metabolites based on MetaMapp and visualized the network to explore biochemical and chemical relationships (Fig. 2C). In this network, nodes with gradient color by FDR-adjusted *P* are NAFLD-specific metabolites. We observed that the network could be divided into 2 main clusters: glycerophospholipid metabolites and BA metabolites.

### Correlation of Metabolites and Clinical Variables

**Figure 3.**
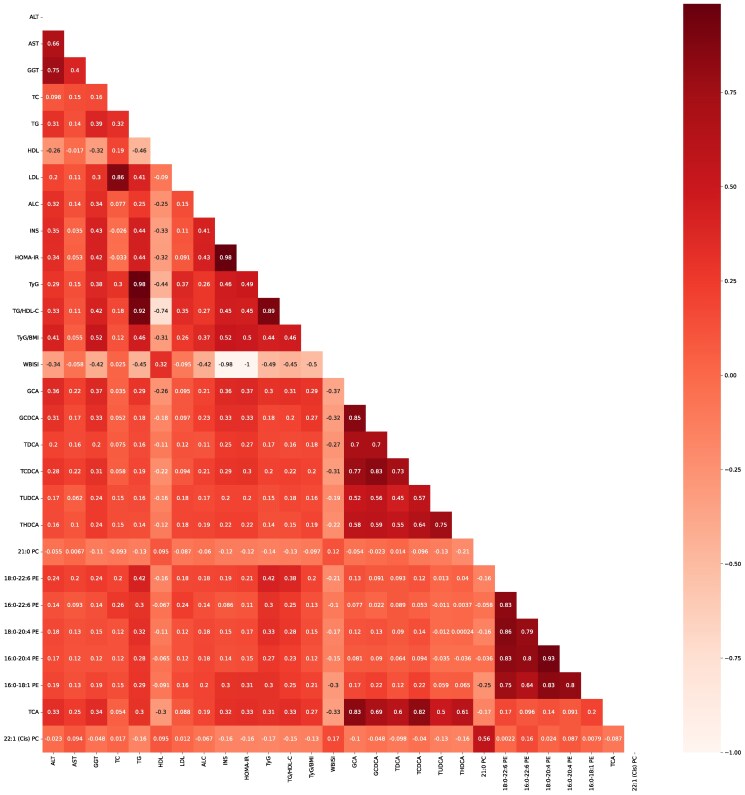
Correlations between NAFLD-specific metabolites and clinical variables. Correlation plots showing Spearman correlation values of NAFLD-specific metabolites and clinical variables.

The 18:0 to 22:6 PE was positively correlated with TC, TyG, and TG/HDL-C. Spearman correlation coefficients were 0.418, 0.422, and 0.380, respectively, with *P* values of .016, <.001, and <.001, respectively (Fig. 3, Supplementary Table S2) [24]. Additionally, GCA was positively correlated with ALT, GGT, INS, and HOMA-IR, Spearman correlation coefficients were 0.363, 0.368, 0.362, and 0.373, respectively, with every *P* value <.001 (Fig. 3, Supplementary Table S2) [24].

### Application of Clinical Variables and Metabolic Characteristics in Developing Interpretable Machine Learning Models

**Figure 4.**
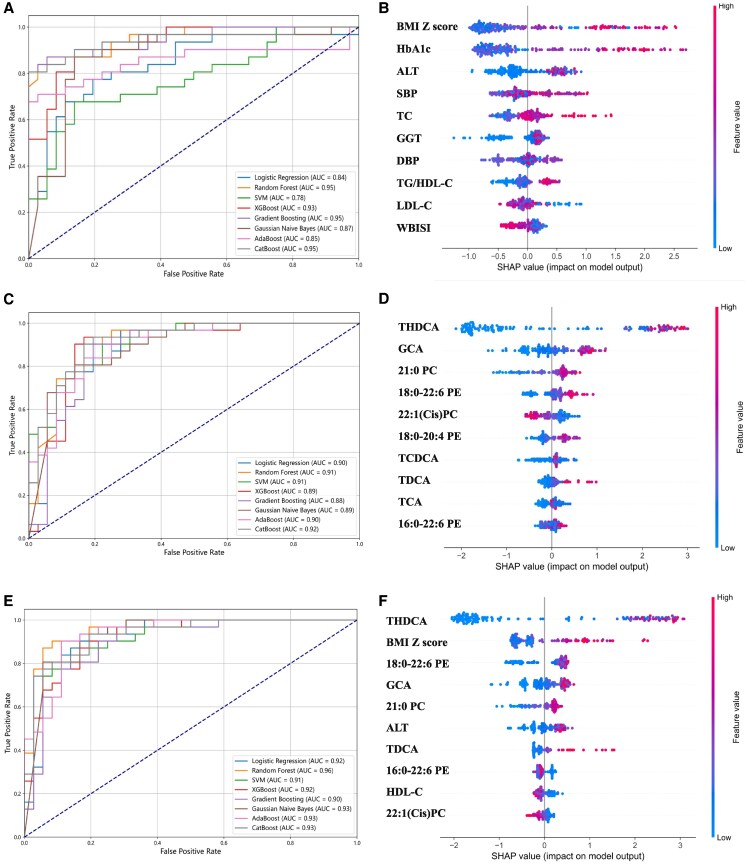
(A, C, E) Receiver operating characteristic analysis showed that clinical variables, metabolites, and clinical variables combined with metabolites could be important variables in the diagnosis model of NAFLD developed by machine learning methods, respectively. (B, D, F) Contribution analysis to the prediction of the random forest model in the training dataset via the SHAP technique for clinical variables, metabolites, and clinical variables combined with metabolites, respectively. Each point is a patient, the higher the ranking is, the more important the characteristics. The point on the left side of the digital baseline (with a SHAP value of 0) represents a negative contribution to suffering from NAFLD, whereas the point on the right represents a positive contribution. The greater the distance from the baseline is, the greater the impact. SHAP, Shapley additive explanations.

We established machine learning diagnostic models using 16 clinical variables first. All 8 diagnostic models showed excellent predictive performance, with AUC values ranging from 0.775 to 0.954 (Fig. 4A, Table 3). According to the random forest model, the SHAP plot indicated that the top 10 important clinical variables were as follows: BMI *Z* score, HbA1c, ALT, SBP, TC, GGT, diastolic blood pressure, TG/HDL-C, LDL-C, and WBISI (Fig. 4B).

**Table 3.** Summary of the performance metrics of the diagnostic model using 8 machine learning methods

	NAFLD-specific metabolites							
	Logistic regression	Random forest	SVM	XGBoost	Gradient boosting	Gaussian-naive Bayes	AdaBoost	CatBoost
AUC	0.899	0.907	0.915	0.887	0.882	0.892	0.897	0.917
Accuracy	0.836	0.821	0.836	0.806	0.821	0.836	0.836	0.806
Sensitivity	0.836	0.821	0.836	0.806	0.821	0.836	0.836	0.806
Precision	0.842	0.855	0.838	0.833	0.843	0.836	0.853	0.833
Specificity	0.917	0.694	0.889	0.694	0.722	0.861	0.750	0.694
F1	0.834	0.819	0.835	0.804	0.820	0.836	0.835	0.804

	Clinical variables							
	Logistic regression	Random Forest	SVM	XGBoost	Gradient boosting	Gaussian-naive Bayes	AdaBoost	CatBoost
AUC	0.841	0.954	0.775	0.929	0.941	0.873	0.854	0.946
Accuracy	0.776	0.910	0.746	0.881	0.881	0.806	0.791	0.881
Sensitivity	0.776	0.910	0.746	0.881	0.881	0.806	0.791	0.881
Precision	0.785	0.912	0.761	0.883	0.881	0.811	0.791	0.881
Specificity	0.889	0.944	0.889	0.861	0.889	0.889	0.833	0.917
F1	0.772	0.910	0.739	0.881	0.881	0.804	0.790	0.88

	Clinical variables and NAFLD-specific metabolites							
	Logistic regression	Random Forest	SVM	XGBoost	Gradient boosting	Gaussian-naive Bayes	AdaBoost	CatBoost
AUC	0.921	0.957	0.913	0.921	0.909	0.935	0.934	0.934
Accuracy	0.851	0.851	0.836	0.836	0.806	0.866	0.866	0.836
Sensitivity	0.851	0.851	0.836	0.836	0.806	0.866	0.866	0.836
Precision	0.851	0.874	0.837	0.845	0.833	0.870	0.875	0.853
Specificity	0.889	0.750	0.833	0.778	0.694	0.833	0.806	0.750
F1	0.850	0.850	0.836	0.836	0.804	0.866	0.866	0.835

Note: The NAFLD model developed based on clinical variables was composed of 16 clinical variables, including BMI *Z* score, SBP, DBP, ALT, AST, GGT, TC, TG, HDL-C, LDL-C, HbA1c, INS, HOMA-IR, TyG, TG/HDL-C, and WBISI. The NAFLD model developed based on NAFLD-specific metabolites was composed of 14 NAFLD-specific metabolites, including 16:0 to 22:6 PE, 18:0 to 22:6 PE, 21:0 PC, tauroursodeoxycholic acid, THDCA, taurocholic acid, tauroursodeoxycholic acid, GCA, glycochenodeoxycholic acid, 22:1 (Cis) PC, 18:0 to 20:4 PE, 16:0 to 20:4 PE, 16:0 to 18:1 PE, and taurine conjugated cholic acid.

Abbreviations: AdaBoost, adaptive boosting; ALT, alanine aminotransferase; AST, aspartate aminotransferase; BMI, body mass index; DBP, diastolic blood pressure; CatBoost, categorical boosting; GGT, γ-glutamyl transferase; HbA1c, glycosylated hemoglobin; HDL-C, high-density lipoprotein cholesterol; HOMA-IR, homeostasis model assessment of insulin resistance; INS, insulin; LDL-C, low-density lipoprotein cholesterol; OR, odds ratio; SBP, systolic pressure; SVM, support vector machine; TC, total cholesterol; TG, triglyceride; TyG, triglyceride-glucose index; URIC, uric acid; WBISI, whole-body insulin sensitivity index; WC, waist circumference; WHtR, waist-to-height ratio; XGBoost, extreme gradient boosting.

We established other machine learning diagnostic models using 14 NAFLD-specific metabolites identified in this study. All 8 diagnostic models showed excellent predictive performance, with AUC values ranging from 0.882 to 0.917 (Fig. 4C, Table 3). According to the random forest model, the SHAP plot indicated that the top 10 important metabolites were as follows: THDCA, GCA, 21:0 PC, 18:0 to 22:6 PE, 22:1(Cis)PC, 18:0 to 20:4 PE, taurocholic acid, TDCA, taurine conjugated cholic acid, and 16:0-22:6 PE (Fig. 4D).

In addition, clinical variables combined with metabolites were used to construct better diagnostic models for NAFLD in obese children. All 8 diagnostic models showed excellent predictive performance, with AUC values ranging from 0.909 to 0.957; the difference of prediction performance among the models was small (Fig. 4E, Table 3). According to the random forest model, the SHAP plot indicated that the top 10 important metabolites were as follows: THDCA, BMI *Z* score, 18:0 to 22:6 PE, GCA, 21:0 PC, ALT, TDCA, 16:0 to 22:6 PE, HDL-C, and 22:1(Cis)PC (Fig. 4F).

## Discussion

NAFLD is replaced by metabolic dysfunction-associated fatty liver disease as the preferred nomenclature in children now [25]. The new name definitively reveals the pathophysiology of fatty liver with metabolic dysfunction and insulin resistance [26]. We still used the name of NAFLD to ensure consistency with existing research and guidelines.

Obese children are always at risk for NAFLD and metabolic syndrome [27]. There are limited studies on the modalities of screening for NAFLD in children, and the sensitivity of ultrasound for NAFLD diagnosis varies. Machine learning algorithms can build complex models to make accurate disease predictions [23]. In this study, we applied multiple machine learning methods using clinical variables and metabolites to build an NAFLD diagnosis. Random forest and categorical boosting outperformed other machine learning methods. Both had the ability to quickly process complex features and high-dimensional data, providing powerful classification capabilities and high-precision predictions [23].

In our study, acanthosis nigricans was associated with NAFLD after adjusting for gender, age, and BMI as confounding factors. But it was not associated with NAFLD, after adjusting for gender, age, BMI, and WBISI. This means that acanthosis nigricans may be a hallmark of NAFLD, reflecting metabolic abnormalities associated with insulin resistance rather than independent risk factors. Previous reports have also shown that acanthosis nigricans usually as a consequence of insulin resistance status [28]. Therefore, we did not include acanthosis nigricans as a clinical variable to identify NAFLD. We had incorporated 16 clinical variables to build an NAFLD diagnostic model; these variables were elevated in the ON group and showed statistical significance in all 3 multivariable binary logistic regression analyses. The optimal variables for all subjects selected were BMI *Z* score, HbA1c, ALT, SBP, WBISI, TG/HDL-C, and TyG. Insulin sensitivity indicators, HOMA-IR and WBISI, were effective predictors of NAFLD [17, 19, 29]. Reduced insulin sensitivity leads to the promotion of liver fat accumulation. Previous studies have shown a significant correlation between WBISI and liver fat content [29, 30]. In this study, it was confirmed that insulin sensitivity indexes were correlated with NAFLD in a nonlinear dose-response manner. The lipid metabolism indexes, TyG and TG/HDL-C, were correlated with NAFLD in a linear dose-response manner. TyG was considered as an important marker for metabolic syndrome in overweight and obese and served as a new marker for NAFLD [31, 32]. Additionally, a higher TG/HDL-C was associated with an increased risk of liver fibrosis in children and adolescents [33]. Previous studies used TyG and TG/HDL alone to assess NAFLD in children, with AUCs of 0.76 and 0.67, respectively [34, 35].

NAFLD diagnosis model based on metabolites has been widely studied in adults [36, 37]. Our study, conducted on obese children, identified 14 plasma metabolites associated with NAFLD, including 7 glycerophospholipid metabolites (5 PE metabolites and 2 PC metabolites) and 7 BAs. Glycerophospholipid metabolites are the main components of cell membrane and play a crucial role in the process of lipid metabolism [38, 39]. In NAFLD, lipid deposition and oxidative stress disrupt the cytidine diphosphate choline pathway, altering the activity of PE methyltransferase and thereby impeding the production of PC or the conversion of PE to PC, ultimately leading to abnormal levels of PE and PC [39]. In the ON group, the ascending metabolites include 16:0 to 20:4 PE, 16:0 to 22:6 PE, 18:0 to 22:6 PE, and 21:0 PC, and the descending metabolites include 16:0 to 18:1 PE, 18:0 to 20:4 PE, and 22:1(Cis)PC. When PE levels are abnormal, it may increase the activity of phospholipase D and decrease the activity of hormone-sensitive lipase, resulting in imbalanced in fat metabolism, which leads to elevated TG levels [40]. TG/HDL-C, another indicator of abnormal lipid metabolism, will also increase [33]. On the other hand, abnormal PE levels may change membrane physical properties of the membrane, affecting the localization and aggregation of insulin receptors on the membrane, aggravating insulin resistance [41, 42]. It is well known that insulin resistance is one of the main factors leading to the rise of TyG. This can also explain our finding that 18:0 to 22:6 PE was positively correlated with TC, TyG, and TG/HDL-C. Abnormal lipid metabolism and insulin resistance exacerbates NAFLD progression [43]. Additionally, abnormal PE was associated with chronic low-grade inflammation, which promotes NAFLD progression [38]. These findings highlight the importance of PE metabolites in predicting NAFLD. The NAFLD machine learning model developed by Khusial et al also supports the importance of PE in predicting NAFLD [44].

Physiologically, BAs promote cholesterol excretion from liver and help maintain cholesterol balance. In patients with NAFLD, insulin resistance increases the activity of 12α-hydroxylase CYP8B1 expression, leading to increased BA synthesis [45-47]. Previous studies have suggested that elevated BA metabolites were related to NAFLD in both adults and children, reflecting the adaptive regulation of metabolic pathways in the liver, indicating early liver dysfunction [48-51]. In the ON group of our study, 7 BA metabolites were elevated. Among them, GCA and THDCA increases were particularly noticeable. GCA is a major primary BA formed from glycine combined with cholic acid and secreted into the intestine through the biliary system. In the gut, GCA is converted to secondary bile acids by 7α-dehydroxylase action of the intestinal flora. Excessive accumulation of BAs has adverse effects on the liver and biliary system, exacerbating the pathological process [52-54]. GGT is an enzyme primarily expressed in the liver and bile duct system, and its elevated activity is often associated with cholestasis and liver cell damage. We found that GCA was positively correlated with GGT, which may be partly due to the accumulation of bile acids in hepatocytes, leading to hepatocyte injury and elevated GGT level [53, 54]. SHAP method interpretation model showed that THDCA was the most important feature for predicting NAFLD. THDCA is a secondary bile acid that formed by primary bile acids in liver by binding to taurine.

In NAFLD, THDCA and other secondary bile acids may be toxic to liver cells at high concentrations [46]. They can promote the damage of cell membrane, leading to fatty degeneration of liver cells and the aggravation of an inflammatory response. THDCA activates immune cells in the liver, such as Kupffer cells and hepatic stellate cells, and promotes the release of inflammatory factors. This inflammatory response may lead to lobular inflammation and balloon formation. THDCA concentration was positively correlated with NAFLD histological changes (steatosis grade, lobular inflammation, ballooning, fibrosis stage) [47, 48]. Kalhan and colleagues analyzed untargeted metabolites to understand the biological characteristics of NAFLD [44]. They discovered that THDCA positively correlates with the progression of NAFLD. By applying machine learning techniques to plasma metabolites like THDCA, they developed a NAFLD model with an accuracy of 92%.

Machine learning models that combine clinical variables and metabolite profiles outperform those based on clinical variables or metabolites alone, demonstrating higher specificity, sensitivity, and AUC, with more consistent performance across different approaches. A machine learning method for NAFLD in children developed by Yunfei et al focused only on clinical features (such as ALT, uric acid, insulin, BMI) and had an AUC of up to 0.77 [55]. A machine learning approach developed by Khusial et al focused only on metabolite features (such as phospholipids and amino acid metabolites) achieved an AUC of 0.86. However, when clinical variables including WC, TG, and WBISI were incorporated, AUC increased to 0.94 [56]. In this study, the machine learning model based on clinical variables combined with metabolic characteristics achieved a maximum AUC of 0.957, with differences in AUC among various NAFLD models being less than 0.05, indicating strong model stability. In addition, clinical indicators offer direct evidence of NAFLD, whereas metabolic indicators reveal the disease's underlying mechanisms. Machine learning models combining both provide a more comprehensive approach to diagnosing NAFLD.

However, this is a single-center study included children in north China only. Ultrasound instead of liver biopsy was used for NAFLD diagnosis because of invasiveness. And we employed a targeted metabolite detection method to ensure accurate qualitative and quantitative detections of metabolites. This method offers limited metabolites coverage compared with nontargeted approaches, potentially leaving metabolites undetected. The diagnostic models based on clinical variables and metabolites demonstrated excellent performance for NAFLD prediction. But, because of the nature of cross-sectional, we cannot establish causal relationships. Further studies are necessary to confirm the mechanisms between these selected metabolites and NAFLD.

## Data Availability

Some or all datasets generated during and/or analyzed during the current study are not publicly available but are available from the corresponding author on reasonable request.

## Abbreviations

AdaBoost: adaptive boosting
ALT: alanine aminotransferase
AST: aspartate aminotransferase
AUC: area under the curve
BA: bile acid
BMI: body mass index
DBP: diastolic blood pressure
CatBoost: categorical boosting
FDR: false discovery rate
FPG: fasting plasma glucose
GCA: glycocholic acid
GGT: γ-glutamyl transferase
HbA1c: glycosylated hemoglobin
HDL-C: high-density lipoprotein cholesterol
HOMA-IR: homeostasis model assessment of insulin resistance
INS: insulin
LDL-C: low-density lipoprotein cholesterol
NAFLD: nonalcoholic fatty live disease
PC: phosphatidylcholine
PE: phosphatidylethanolamine
SBP: systolic pressure
SHAP: Shapley additive explanation
TDCA: tauroursodeoxycholic acid
THDCA: taurohyocholic acid
TyG: triglyceride-glucose index
WBISI: whole-body insulin sensitivity index
WC: waist circumference
